# Effects of hydrotherapy and Hammock positioning on weight gain in low-birth-weight premature newborns: a randomized clinical trial

**DOI:** 10.1016/j.jped.2025.03.005

**Published:** 2025-04-19

**Authors:** Jeferson de Sousa Justino, Francisco Plácido Nogueira Arcanjo, Luiz Odorico Monteiro de Andrade, Ivana Cristina de Holanda Cunha Barreto, Lizandro de Andrade Teles, Ana Talita Vasconcelos Arcanjo, Edcley Sousa Teixeira, Marya Clara Barros Mororó

**Affiliations:** a Universidade Federal do Ceará, Programa de Pós-Graduação em Ciências da Saúde, Av. Cmte. Maurocélio Rocha Pontes, 100, Sobral - CE CEP 62042-250, Brazil; b Fundação Oswaldo Cruz, Saúde Pública, Eusébio, CE, Brazil; c Fundação Oswaldo Cruz, Pediatria, Rua São José SN, Eusébio - CE CEP 61773-220, Brazil; d Universidade Federal do Ceará, Faculdade de Medicina, Sobral, CE, Brazil

**Keywords:** Infant, premature, Infant, low birth weight, Hydrotherapy, Weight gain, Hammock positioning

## Abstract

**Objective:**

To evaluate and compare whether hydrotherapy and hammock positioning techniques assist in the weight gain of premature newborns.

**Methodology:**

A single-blind randomized clinical trial was conducted at Santa Casa de Misericórdia Hospital in Sobral, between July 2022 and October 2023. The trial included 16 months of data collection and involved seventy-six premature newborns with low birth weight, of which only sixty were included after meeting the inclusion criteria. These newborns were randomly divided into four groups: one control group and three groups that received different intervention techniques (hydrotherapy, hammock positioning, and a combined group of hydrotherapy and hammock positioning). The newborns were monitored for clinical stability and specific signs before and after the interventions. The techniques were applied daily for 15 days.

**Results:**

During the 15-day follow-up, the control group had a mean weight gain of 305 g. The group that received hydrotherapy gained 346 g, but without significance. The hammock group and the combined hydrotherapy and hammock group showed significant weight gains: the hammock group had an increase of 360 g (*p* = 0.011), while the combined group achieved a gain of 616 g (*p* = 0.0001). Significant increases in arm circumferences were observed in the hammock group and the hydrotherapy combined with the hammock positioning group.

**Conclusion:**

The results indicate that the combination of hydrotherapy and hammock positioning leads to a significant increase in weight gain in premature newborns. The isolated use of hammock positioning also showed positive effects, but the isolated use of hydrotherapy did not yield significant results (Brazilian Registry of Clinical Trials RBR-6 g5f4jz).

## Introduction

Premature and low birth weight newborns are those with a gestational age of <37 weeks and a weight below 2500 g, both of which are often associated.^1^ This association represents a global health challenge, as the complications of prematurity and low birth weight directly impact the majority of neonatal morbidities and mortalities.^2^ Recent data indicate that annually, around 15 to 30 million newborns worldwide are born premature and/or with low birth weight.^3^^,^^4^

Due to metabolic, feeding, and body temperature regulation difficulties, premature newborns with low birth weight are at ten times higher risk of morbidity and mortality compared to full-term newborns with normal weight for their age.^3^ The precise targeting of interventions in neonatal units can provide specialized and comprehensive care for premature and low birth weight newborns, contributing to the reduction of infant morbidity and mortality rates.^5^

In the assessment of growth in premature newborns with low birth weight, weight gain is one of the most important markers for newborn development.^6^ However, the low nutritional reserves acquired in the intrauterine environment, along with exposure to numerous stressors—including monitoring equipment sounds, lights, painful invasive procedures, maternal separation, and other stressful factors—contribute to lower weight gain.^7^ Newborns under stress develop hemodynamic and respiratory instabilities, increasing oxygen consumption and reducing the number of calories available for growth and development.^8^

The complexity of maintaining humanized and quality care for low-birth-weight premature newborns during hospitalization has been a concern for professionals in the field, as they have recognized the need for techniques and therapeutic measures that could minimize the negative impact of prematurity, promoting better quality of life and weight gain.^9^^,^^10^ Given the unfavorable physiological and environmental characteristics of low-birth-weight premature newborns,^11^ hydrotherapy and hammock positioning techniques are being used during hospitalizations, aiming to promote relaxation and reduce stress, with the potential to decrease energy expenditure by simulating intrauterine characteristics such as a warm liquid environment and a flexed posture with space limitation.^12^

Hydrotherapy applied in the neonatal hospital environment involves immersing newborns up to the level of the sternal notch in warm water in plastic or wooden containers, for a predetermined time, using specific movements or in a static manner.^13^^,^^14^ Hammock positioning involves placing newborns inside a cotton fabric sling, which is installed inside incubators or heated cribs.^10^^,^^15^ This technique has been used in the northeastern region of Brazil; however, there are few randomized clinical trials that have evaluated its use as a therapeutic or prophylactic method.^16^

Currently, there are studies indicating that these techniques can reduce the high metabolic demands of newborns, generated by the stress of the extrauterine environment and the pain from manipulative procedures, by inducing sleep and neuromuscular relaxation, thereby facilitating greater weight gain.^13^^,^^14^

Thus, the main objective of this study is to evaluate and compare the weight gain of low-birth-weight premature newborns subjected to hydrotherapy, hammock positioning, and hydrotherapy combined with hammock positioning techniques, comparing them to a control group.

## Material and methods

### Ethical considerations and participant care

The Research Ethics Committee for Human Subjects approved this study (CAAE 89,955,118.3.0000.8109), and it was registered in the Brazilian Registry of Clinical Trials (ReBEC) RBR-6 g5f4jz. A single-blind randomized clinical trial was conducted at *Santa Casa de Misericórdia* Hospital in the city of Sobral, in the state of Ceará. Three techniques (hydrotherapy, hammock positioning, and hydrotherapy combined with hammock positioning) were evaluated and compared with a control group. The application of the techniques and data collection took place from July 2022 to October 2023.

In accordance with Resolution N°. 466/12 of the Brazilian National Health Council (CNS), this study ensured immediate and emergency assistance to research participants whenever needed. Comprehensive care was provided to address complications, adverse events, or any harm resulting directly or indirectly from the study.

Participants did not incur any costs related to this research. In the event of complications such as hypothermia, hyperthermia, water ingestion through nasal, oral, or auditory passages, cross-contamination, hemodynamic instability, asphyxia, or any other adverse events, the participant received immediate care from the principal investigator, the medical team, and the multidisciplinary staff of the Neonatal Intensive Care Unit at Santa Casa de Misericórdia de Sobral.

Included were premature newborns without neurological alterations or genetic syndromes, hospitalized in the Neonatal Intensive Care Unit (NICU) and the Neonatal Intermediate Care Unit (NINTERCU), with a gestational age of <37 weeks, a birth weight of <2500 g, and over 5 days of life. All were clinically stable, with free breastfeeding and/or receiving hydrolyzed milk, and were given a complete enteral diet via an orogastric tube, administered every 3 h.

Premature newborns who did not complete the 15 days of therapy due to hospital discharge, feeding intolerance, clinical instability during the intervention days, use of invasive or non-invasive mechanical ventilation, or use of oxygen, as well as those with neurological alterations, genetic syndromes diagnosed during the study, gastrointestinal disorders such as diarrhea or vomiting for three consecutive days, fasting during oral feeding or breastfeeding, congenital infectious diseases, receiving phototherapy, with peripheral central venous access, or who underwent any surgery during the application of the techniques or data collection were excluded.

During the 16 months of data collection and application of the techniques, seventy-six premature newborns with low birth weight were eligible, as they met the inclusion criteria. Of these, sixteen were excluded during the application of the techniques and data collection due to meeting the exclusion criteria. Thus, 60 premature newborns with low birth weight comprised the study population and were randomized using the Research Randomizer software (https://www.randomizer.org/) for allocation into 4 groups with 15 participants each.

**Figure 1:**
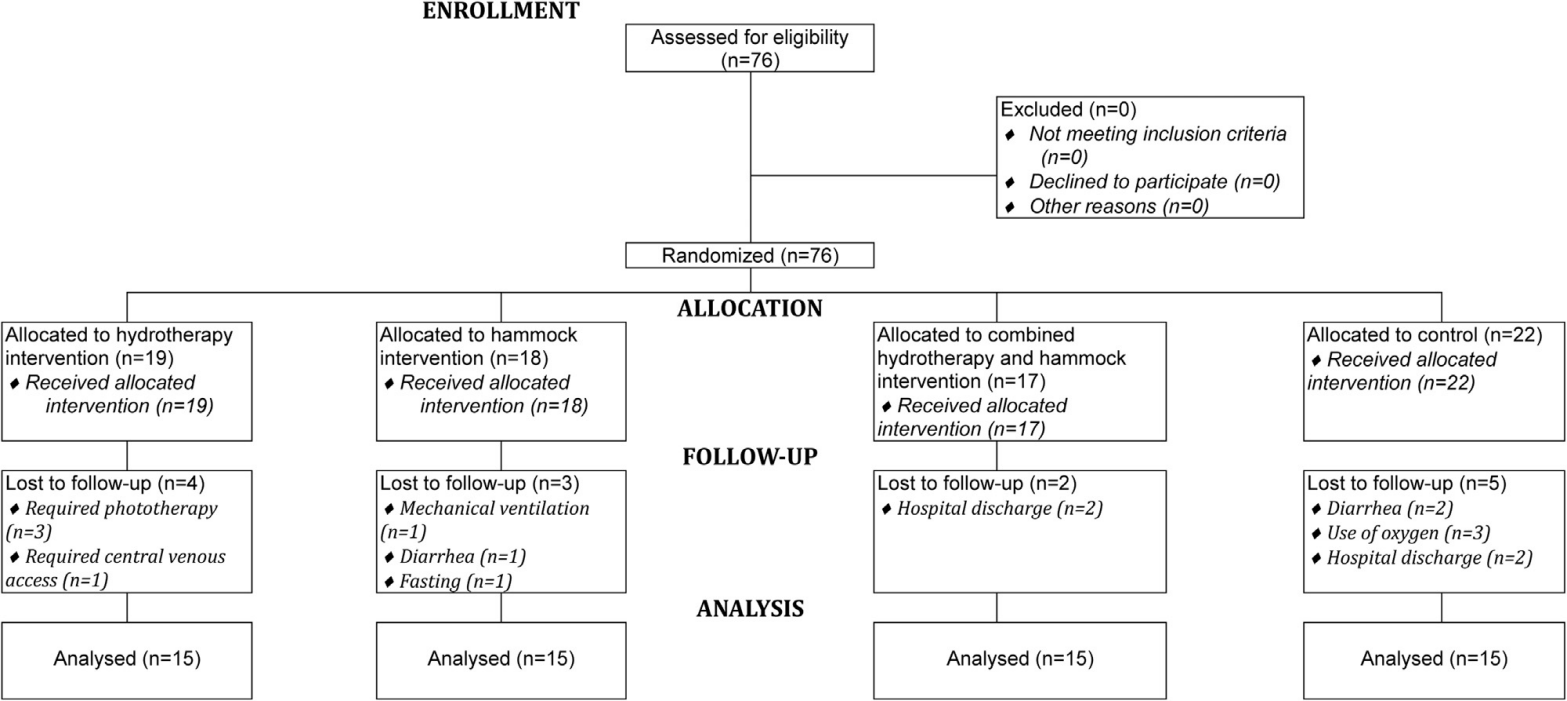
Flow diagram.

Fifteen newborns were allocated to the control group, fifteen to the group that used only the hydrotherapy technique, fifteen to the group that used only the hammock positioning technique, and finally, fifteen formed a group that combined hydrotherapy and hammock positioning techniques. The newborns in the control group received care from the on-duty professional teams, without receiving complementary therapies during the 15-day follow-up. The techniques were applied, and data were collected daily, once a day, for a consecutive period of 15 days (Figure 1).

The composition of the groups, the application of the techniques, and the data collection were conducted gradually due to the insufficient number of eligible newborns at the same time.

Four experienced physical therapists collaborated in the application of the techniques and data collection over the 16 months. These professionals underwent three days of training to replicate the protocol adopted for the techniques during the applications and data collection. The techniques were applied in 15-minute sessions for the hydrotherapy group, 120 min for the hammock positioning group, and 135 min for the combined hydrotherapy and hammock positioning group. The control group received only routine care from the inpatient unit.

Before and after the application of the techniques, the groups were assessed for the safety and stability of vital signs and hemodynamics, measuring heart rate, blood pressure, respiratory rate, peripheral oxygen saturation, axillary temperature, and pain using the Neonatal Facial Coding System (NFCS).

The application of the techniques began in the hospital unit. If the newborn was transferred to another hospital department, the researchers accompanied them until the completion of the 15 days of technique application and data collection, except in cases of hospital discharge.

**Figure 2:**
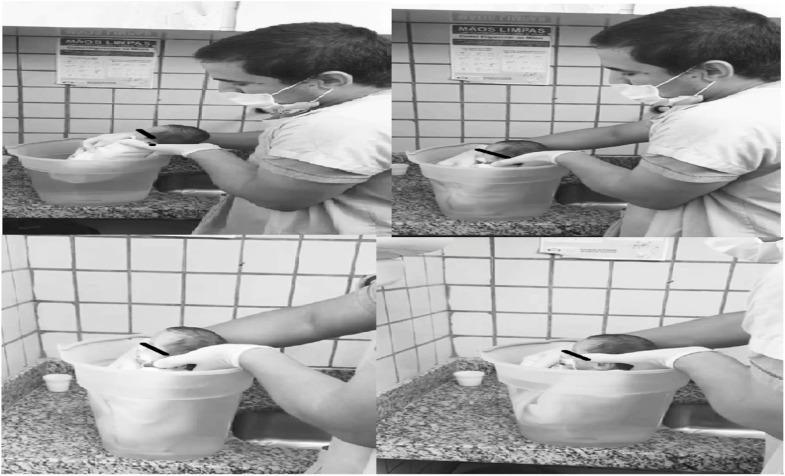
Application of hydrotherapy. Source: author's file.

The newborns participating in the study received their sessions in their respective hospital units. After the preliminary assessment, the newborns undergoing hydrotherapy (Figure 2) were wrapped in a one square meter cotton fabric (towel) in a flexed position of the upper and lower limbs, simulating the position adopted in the intrauterine environment. They were then immersed up to the sternal notch in water heated to 37 ° Celsius, in a 15-liter plastic container, for a period of 15 min. When immersing the newborn in the water, the researcher placed one hand on the cervical region at the level of the mastoid processes and the other hand on the sacral region, positioning the newborn at the bottom of the container as if seated. After securing the newborn at the bottom of the container, the researchers gently positioned their hands between the newborn's jaw and the cervical-occipital region, allowing free movement while keeping the head out of the water. After 15 min, the newborns were removed from the water and the cotton fabric that enveloped them. They were then manually dried with a cotton towel and placed in the incubators or cribs they were using.

The containers used for hydrotherapy were cleaned daily with mild soap before use. These containers measured fifty centimeters in length and forty centimeters in width. A hydrotherapy protocol without movements was adopted, with only immersion up to the sternal notch.

The material used for the hammock was made of 100% cotton fabric, in a rectangular shape, measuring three millimeters in thickness, fifty centimeters in width, and sixty-five centimeters in length.

**Figure 3:**
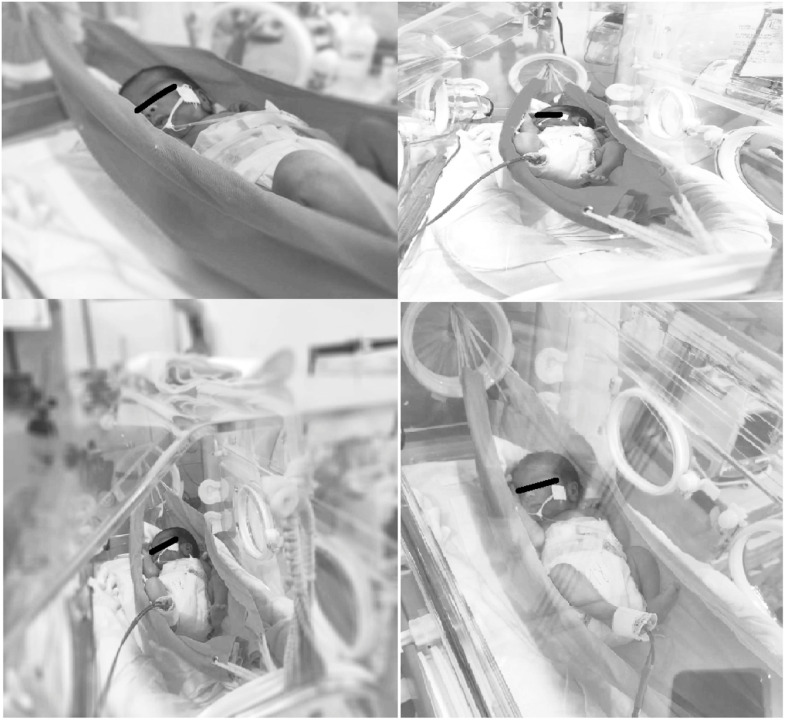
Application of hammock positioning. Source: author's file.

The hammock positioning (Figure 3) was performed inside the incubators, ten centimeters above the mattress, with the ends tied and secured externally. The newborns were placed in a supine position for 120 min, with a fabric placed under the scapular and cervical regions to prevent excessive neck flexion. For safety, the orogastric tubes of the newborns using them were left open, and pulse oximetry was used throughout the therapy to continuously measure peripheral oxygen saturation and pulse. After the allotted time, the participants were returned to the incubator and remained under the care of the on-duty professional team.

During the study, seven hammocks were used for the application of the techniques; all of them were washed and disinfected every five days of use at the hospital's own laundry or whenever the sample changed.

The newborns in the combined hydrotherapy and hammock positioning group were first subjected to the hydrotherapy technique. After the 15-minute session, they were manually dried with towels and placed in the hammock position for 120 min, following the same protocol already described.

The outcomes measured included the weight and arm circumference of the newborns, which were assessed daily for 15 days. Weight was measured using the Balmak® pediatric scale (Model Mobile Baby ELP 25BB) (calibrated daily using a standard weight technique before weighing the newborns), while arm circumference was recorded with an anthropometric measuring tape. All data were documented on specific forms.

### Statistical analysis

The statistician received data from each group, labeled with numbers 1 through 4, without any identification of which intervention each number corresponded to (Group 1: hydrotherapy, Group 2: hammock positioning, Group 3: combined hydrotherapy and hammock positioning, and Group 4: control). The group identities were only revealed after the statistical analysis was completed.

The organization and tabulation of the data were performed using Microsoft Office Professional Plus Excel 2016 (Microsoft Corp., United States). The Statistical Package for the Social Sciences (SPSS) software, version 22 (International Business Machines Corporation, Armonk, NY, USA), was used for data analysis. In order to verify whether the mean weight gain and increase in arm circumference differed across the three interventions (hydrotherapy, hammock positioning, and the combined use of hydrotherapy and hammock positioning) compared to the control group, specific statistical analyses were conducted. Quantitative variables were expressed as mean and standard deviation. Statistical tests were conducted with a 95% confidence interval (CI), and a significance level of *p* < 0.05 was considered. Data distribution was assessed for normality using the Shapiro-Wilk test. The comparison of paired and unpaired quantitative variables was conducted using the Student's *t*-test.

## Results

Baseline variables such as gestational age in weeks, age in days of the newborns at the start of the interventions, birth weight in grams, weight at the start of the interventions, days of antibiotic use, days of hospitalization, and days of mechanical ventilation showed no statistical differences across the three study groups when compared to the control group, indicating homogeneous groups for comparative testing (Table 1).

**Table 1:** Baseline variables of the study groups.

**Baseline variables**	**Control group**	**Hydrotherapy group**		**Hammock group**		**Hydrotherapy and hammock group**	
	M^a^ ± SD^b^	M^a^ ± SD^b^	p^c^	M^a^ ± SD^b^	p^c^	M^a^ ± SD^b^	p^c^
Gestational age in weeks	31.4 ± 3.29	32.4 ± 2.26	0.309	31.4 ± 2.64	0.951	31.6 ± 2.29	0.848
Age at the start of the intervention in days	13.1 ± 10.06	11.5 ± 10.95	0.680	11.7 ± 10.23	0.708	12.1 ± 11.35	0.813
Birth weight in grams	1653 ± 373.3	1721 ± 404.6	0.636	1636 ± 380.2	0.901	1649 ± 471.6	0.976
Weight at the start of the intervention	1580 ± 464.4	1780 ± 395.7	0.192	1651 ± 379.1	0.650	1514 ± 428.8	0.772
Days of antibiotic use	21.4 ± 7.14	15.8 ± 11.76	0.486	17.9 ± 16.89	0.682	12.6 ± 9.27	0.263
Days of hospitalization	36.9 ± 15.55	33.5 ± 11.43	0.492	38.0 ± 23.87	0.885	37.3 ± 25.56	0.959
Days of mechanical ventilation use	5.0 ± 0.48	6.0 ± 0.50	>0.999	3.0 ± 0.41	0.681	5.0 ± 0.48	>0.999

a Mean.

b Standard deviation.

c *p* value calculated using the unpaired Student's *t*-test.

During the 15 days of follow-up, the control group showed a mean weight gain of 305 g and a mean increase of 0.23 cm in brachial circumference. The hydrotherapy group experienced greater weight gain and an increase in brachial circumference compared to the control group, but without statistical significance, with a mean weight gain of 346 g (*p* = 0.132) and a mean increase in brachial circumference of 0.34 cm (*p* = 0.057). The hammock group and the combined hydrotherapy and hammock group had a significant increase in mean weight when compared to the control group, with gains of 360 g (*p* = 0.011) and 616 g (*p* = 0.0001), respectively. The mean gain in brachial circumference was 0.40 cm (*p* = 0.003) in the hammock group and 0.66 cm (*p* = 0.0001) in the combined hydrotherapy and hammock group. The combined hydrotherapy and hammock positioning group showed the greatest weight gain and brachial circumference gain compared to all groups, with statistically significant values relative to the control group (Table 2).

**Table 2:** Effect of the interventions on weight gain and brachial circumference.

**Variables**	**Control group**	**Hydrotherapy group**		**Hammock group**		**Hydrotherapy and hammock group**	
	M^a^ ± SD^b^	M^a^ ± SD^b^	*p*^c^	M^a^ ± SD^b^	*p*^c^	M^a^ ± SD^b^	*p*^c^
Mean weight gain in grams	305 ± 44.2	346±91.5	0.132	360 ± 65.0	0.011	616 ± 91.6	0.0001
Mean brachial circumference in centimeters	0.23 ± 0.10	0.34±0.18	0.057	0.40 ± 0.18	0.003	0.66 ± 0.20	0.0001

a Mean.

b Standard deviation.

c *p* value calculated using the unpaired Student's *t*-test.

## Discussion

This study compared the impact of hydrotherapy, hammock positioning, and the combined use of these two techniques on weight gain in low-birth-weight premature newborns with a control group. In the present study, it was possible to observe that the hydrotherapy technique influenced the increase in weight gain among the newborns, even though it did not show statistical significance in the results compared to the control group.

Results similar to the present study were found in research conducted by Lemos et al.,^17^ which did not include a control group and had few interventions and follow-ups. They investigated the effects of a hydrotherapy technique after two weekly sessions of 10 min each on the relaxation and weight gain of 10 clinically stable premature newborns, finding no significant increase in weight gain (*p* = 0.127). However, the study by Silva et al.,^18^ using the same methodology but with 30 stable premature newborns, found a significant weight gain over the two days of intervention (*p* < 0.001), increasing from 1983 g in the first session to 2044 g in the second session.

Anjos et al.^19^ conducted a study similar to ours, but without a control group and with only five applications. They worked with forty-four randomized premature newborns divided into two groups, comparing hydrotherapy with heated water and tactile-kinesthetic stimulation regarding weight gain in premature infants hospitalized in the Neonatal Intensive Care Unit (NICU). This study found greater weight gain in the hydrotherapy group compared to the tactile-kinesthetic stimulation group.

It is possible that the weight gain observed in some studies following the use of hydrotherapy with heated water may be related to the maintenance of clinical stability, reduction of stress, and improvement in sleep quality. This could lead to decreased sensory overload and relaxation, potentially associated with a possible reduction in cortisol levels.^12^^,^^14^ The study by Tobinaga et al.^13^ found a significant reduction in salivary cortisol (*p* = 0.004) after a hydrotherapy application in 15 premature newborns, which may relate to a possible decrease in metabolism and hormone levels associated with stress, resulting in reduced activation of the sympathetic nervous system. This promotes a state of relaxation characterized by regular breathing, decreased muscle tone, and improved sleep in these newborns.

The mechanism behind weight gain in newborns who undergo hydrotherapy, as reported in some studies, remains unclear. It is important to note that, even in clinically stable premature infants, the physiological stress caused by interventions and manipulations can increase energy expenditure, potentially leading to weight loss. This may help explain why the hydrotherapy group in this study showed no significant difference in weight gain compared to the control group.

Some reviews link the use of the hammock position in hospitalized newborns with increased sleep duration and quality, reduced pain, and stress, improved neuromuscular development, and stabilization of vital signs.

As far as the authors could identify in this review, we found one study in the literature that addresses the relationship between the use of hammock positioning and weight changes. This pioneering clinical trial, conducted with twenty very low birth weight premature infants randomized into two groups, compared hammock positioning with the nested prone position, a traditional position currently used in neonatal admissions. The researchers conducted hammock positioning sessions for three hours daily over ten consecutive days. Compared to the nested prone position, the hammock position was associated with a higher neuromuscular maturity score (*p* < 0.003) and a more relaxed condition, indicated by lower heart and respiratory rates (*p* < 0.05 and *p* < 0.01, respectively); however, weight gain did not differ between the groups during the ten days of follow-up (*p* < 0.1).^10^

A more recent study with twenty-six clinically stable newborns, gestational age of 30 to 37 weeks, compared the use of hammock positioning and cotton nest positioning concerning sleep, pain, and vital signs over five days with one application per day. Individuals in the hammock positioning group showed improved pain (*p* = 0.008), increased sleep duration and quality (*p* < 0.001), and decreased vital signs while maintaining physiological parameters (*p* < 0.001) compared to the traditional cotton nest positioning group.^20^

Costa et al.,^16^ in a randomized cross-over clinical trial with twenty premature newborns, found statistical significance with the use of hammock positioning in favor of the sleep of newborns compared to its non-use. Another clinical trial conducted in northeastern Brazil, also with a sample of 20 premature newborns admitted to the NICU, observed significant improvements in vital signs and pain after 40 min of hammock positioning (*p* < 0.05).^21^

Another study with 28 premature infants between 28 and 36 weeks of gestational age, conducting one application for 60 min, revealed that the use of hammock positioning does not decrease pain scores (*p* = 0.42) but induces sleep more rapidly (*p* < 0.001) and reduces vital signs while maintaining physiological normality (*p* < 0.001).^22^ In contrast, a study with a sample of 8 premature newborns, performing three daily applications, found a statistically significant difference (*p* < 0.05) in pain reduction and decreased heart and respiratory rates, while maintaining physiological normality after using the hammock position for two hours.^23^

Based on the present study and some of the clinical trials mentioned above, it is believed that the use of hammock positioning as a therapeutic measure to assist in weight gain for premature newborns may be related to an increase in hours of sleep and a decrease in stress and painful stimuli that elevate energy expenditure in this age group. As noted by Lyngstad et al.,^24^ high levels of stress in the neonatal hospital setting can result in lasting effects, heightening the child's sensitivity to pain and stress and subsequently raising energy expenditure. It is known that stressful physical stimuli present in neonatal intensive care units, such as routine repeated manipulations during hospitalization, evoke pain responses due to the immature nociceptive system of the premature newborn.^16^^,^^25^ Thus, the state of relaxation provided by hammock positioning could help prevent unnecessary energy expenditure.

Premature newborns possess all the functional and neurochemical components necessary for transmitting and receiving pain signals. However, their responses to stimuli are nonspecific and disorganized due to the incomplete myelination of the nervous system and the immaturity of their endogenous pain control systems, which modulate pain. In this regard, the pain experienced by these newborns is much greater and more acute than that felt by adults, leading to physical and psychological discomfort and suffering for these infants.^26^

The increase in weight gain observed in the clinical trial following the use of hydrotherapy and hammock positioning, along with reports from current literature, suggests that this increase in weight gain may have multifactorial relationships. These include the reduction of cortisol levels, decreased pain and stress, and improved sleep duration and quality, all of which directly reflect a decrease in unnecessary energy expenditure beyond basal metabolism.

The results of this study show that the combined use of hydrotherapy and hammock positioning significantly promotes weight gain in hospitalized premature newborns. The isolated use of hammock positioning also demonstrated statistically significant positive effects on the weight gain of these newborns. Although isolated hydrotherapy did not show statistically significant results in weight gain, it can be considered a complementary resource to assist in this process. These non-pharmacological techniques appear to be safe, simple, and cost-effective for use in premature newborns during hospitalization.

## Funding

None.

## Conflicts of interest

The authors declare no conflicts of interest.
